# The effects of PI3K-mediated signalling on glioblastoma cell behaviour

**DOI:** 10.1038/s41389-017-0004-8

**Published:** 2017-11-29

**Authors:** Julia Langhans, Lukas Schneele, Nancy Trenkler, Hélène von Bandemer, Lisa Nonnenmacher, Georg Karpel-Massler, Markus D. Siegelin, Shaoxia Zhou, Marc-Eric Halatsch, Klaus-Michael Debatin, Mike-Andrew Westhoff

**Affiliations:** 1 Department of Pediatrics and Adolescent Medicine, University Medical Center Ulm, Ulm, Germany; 2 Department of Pathology and Cell Biology, Columbia University Medical Center, New York, NY USA; 3 Department of Neurosurgery, University Medical Center Ulm, Ulm, Germany; 4 Department of Clinical Chemistry, University Medical Center Ulm, Ulm, Germany

## Abstract

The PI3K/Akt/mTOR signalling network is activated in almost 90% of all glioblastoma, the most common primary brain tumour, which is almost invariably lethal within 15 months of diagnosis. Despite intensive research, modulation of this signalling cascade has so far yielded little therapeutic benefit, suggesting that the role of the PI3K network as a pro-survival factor in glioblastoma and therefore a potential target in combination therapy should be re-evaluated. Therefore, we used two distinct pharmacological inhibitors that block signalling at different points of the cascade, namely, GDC-0941 (Pictilisib), a direct inhibitor of the near apical PI3K, and Rapamycin which blocks the side arm of the network that is regulated by mTOR complex 1. While both substances, at concentrations where they inhibit their primary target, have similar effects on proliferation and sensitisation for temozolomide-induced apoptosis, GDC-0941 appears to have a stronger effect on cellular motility than Rapamycin. In vivo GDC-0941 effectively retards growth of orthotopic transplanted human tumours in murine brains and significantly prolongs mouse survival. However, when looking at genetically identical cell populations that are in alternative states of differentiation, i.e. stem cell-like cells and their differentiated progeny, a more complex picture regarding the PI3K/Akt/mTOR pathway emerges. The pathway is differently regulated in the alternative cell populations and, while it contributes to the increased chemo-resistance of stem cell-like cells compared to differentiated cells, it only contributes to the motility of the latter. Our findings are the first to suggest that within a glioblastoma tumour the PI3K network can have distinct, cell-specific functions. These have to be carefully considered when incorporating inhibition of PI3K-mediated signals into complex combination therapies.

## Introduction

The results of several recent clinical evaluations suggest that personalised medicine, targeting tumour-specific alterations in signalling cascades, rarely improves outcomes compared to standard therapy, i.e. doctors' choice^1^. These findings are surprising, as these alterations are the underlying molecular basis for the so-called hallmarks of cancer^2,3^. Targeting activated pathways in malignant cells should therefore block the advantage cancer has over its environment and thus sensitise them for treatment.

Among the most commonly altered signalling cascades in cancer is the PI3K/Akt/mTOR survival network^4^. Despite its complexity, it is frequently considered a promising therapeutic target^5^, although this intensive research into harvesting the therapeutic potential of PI3K/Akt/mTOR-specific pharmacological inhibitors has not been satisfactory, with—so far—only one PI3K (class I) inhibitor, Idelalisib, being approved for cancer therapy^6^. The situation for substances blocking mTOR, or dual-kinase inhibitors, concurrently targeting PI3K and mTOR is not much better^7,8^.

A tumour entity with particularly high rates of active PI3K signalling is glioblastoma, where various alterations result in an aberrant activation of this signalling cascade in ~88% of all cases^9,10^. However, unlike for example in Hodgkin's Lymphoma^11^, inhibition of PI3K-mediated signals does not lead to apoptosis per se in glioblastoma, suggesting that these brain tumours are not oncogenically addicted to this pathway.

Glioblastoma, also known as glioblastoma multiforme, is a World Health Organization-classified grade IV astrocytoma, i.e. the most malignant grade of gliomas^12^. Comprising ~25% of all brain tumours in adults^13^ with patients showing an average life expectancy of only ~1 year, glioblastoma is the most common, as well as most lethal, primary brain tumour in adults^14^.

Our own recent data with the dual-kinase inhibitor PI-103, blocking both PI3K and mTOR, suggest that the PI3K signalling cascade plays a role in regulating the motility of differentiated glioblastoma cells, while only having a marginal effect on their survival upon simple combination treatment with a chemotherapeutic^15^. This is of particular interest, as among the foremost cellular aspects that make glioblastoma highly lethal and difficult to treat is the tumour's ability to grow diffusely and highly invasively, infiltrating the surrounding brain tissue and thus making localised treatment, e.g. surgery, particularly ineffective^16^.

The PI3K network is a highly complex signalling cascade, distinct arms of which regulate diverse cellular processes, such as motility, survival and proliferation^17^. In the present study, we aim to elucidate further the role of this network in glioblastoma. Therefore, we block PI3K-mediated signalling at two distinct key points of the signalling cascade, near apical at the level of PI3K or considerably further downstream at the level of mTOR complex 1 and investigate what effects these interventions have on various aspects of cellular behaviour, such as apoptosis sensitivity and motility.

## Results and discussion

### The effects of PI3K and mTOR inhibition on established glioblastoma cell lines

**Fig. 1:**
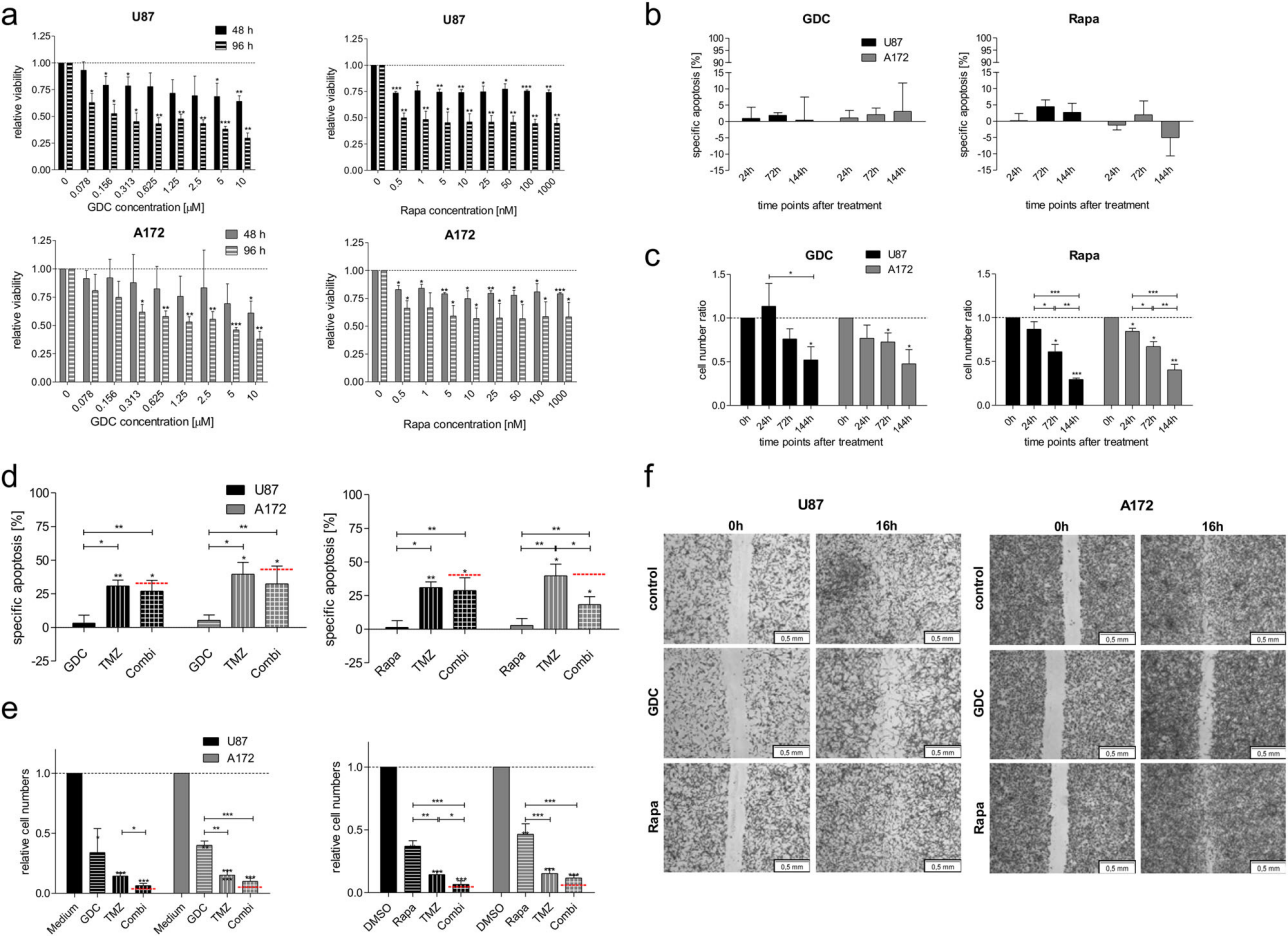
GDC-0941 (Pictilisib) and Rapamycin affect PI3K-mediated signalling in glioblastoma cell lines **a** The effect of the indicated concentrations of the two pharmacological inhibitors, GDC-0941 from Selleckchem (Munich, Germany) (GDC) left and Rapamycin from Sigma-Aldrich (Hamburg, Germany) (Rapa) right, on the viability of two glioblastoma cell lines, U87 (upper panel) and A172 (lower panel). Viability was assessed by MTT assay, while cell lines were obtained from ATCC (Manassas, VA, USA) and cultured as previously described^15,40^. **b** Specific DNA fragmentation was used as surrogate readout for apoptosis as assessed by flow cytometric analysis of propidium iodide-stained nuclei, as previously described^40^, for up to 144 h treatment with either 0.6 μM GDC-0941 (left) or 5 nM Rapamycin (right). **c** Relative changes in cell numbers (control set at 1 for each individual time point), for up to 144 h treatment with either GDC-0941 (left) or Rapamycin (right). Cells were counted using CASY1 DT (Innovatis, Reutlingen, Germany), as previously described^26^. **d** Specific DNA fragmentation used as surrogate readout for apoptosis as assessed by flow cytometric analysis of propidium iodide-stained nuclei, for a 144 h treatment with either 0.6 μM GDC-0941 (left) or 5 nM Rapamycin (right) and 100 μM temozolomide from Sigma-Aldrich (TMZ) and a combination of either inhibitor with TMZ (Combi). Red line indicates additive effect, i.e. below red line: antagonism, above red line: synergism. **e** Relative changes in cell numbers (solvent control set at 1), for a 144 h treatment with either 0.6 μM GDC-0941 (left) or 5 nM Rapamycin (right) and 100 μM temozolomide (TMZ) and a combination of either inhibitor with TMZ (Combi). Red line indicates additive effect, i.e. below red line: synergism, above red line: antagonism. **f** U87 (left) and A172 (right) cells were incubated with 0.6 μM GDC-0941, 5 nM Rapamycin or solvent (control). Scratches were introduced 1 h after treatment initiation and from then semi-directional cell migration was examined after 16 h by fixation of the cells and subsequent staining, as previously described^15^. Scale bars equal 500 μm. Experiments shown in **a** were performed at least three times in sextet, shown are mean and +SD, while **b**–**e** show results of at least three independent experiments performed in triplicate, shown are mean and +SD. An unpaired two-tailed *t* test was used for statistical evaluation (*p < 0.05, **p  < 0.01, ***p < 0.001). In **f**, one of two representative independent experiments performed in triplicate is shown

Two established glioblastoma cell lines, U87 and A172, were selected neither of which express PTEN, the negative regulator of PI3K^18^. These cell lines were treated with either GDC-0941 (Pictilisib), which binds to PI3K in an ATP-competitive manner, or Rapamycin which is a bacterially derived natural mTOR inhibitor^19,20^. Both substances have similar effects on viability, spontaneous apoptosis and total cell numbers (Fig. 1a–c). However, an ~100-fold higher concentration of GDC-0941 was needed to achieve similar results as with Rapamycin, i.e. 625 nM compared to 5 nM (Fig. 1a). Interestingly, while no additive effects with increasing concentrations of Rapamycin could be observed, this was not the case for GDC-0941, which displayed a superior effect on cell viability at high concentrations (Fig. 1a). While glioblastoma cell lines can be sensitised for apoptosis induction via both chemotherapy and death ligands by a combination of PI3K/mTOR inhibition and serum withdrawal^21,22^, combining just PI3K/mTOR inhibition with chemotherapy in glioblastoma cell lines can actually reduce cell death depending on the sequence of applications given^23^. Inhibition of either PI3K or mTOR has a strong effect on cell numbers (Fig. 1c), but—as already suggested by our previous work with the dual-kinase inhibitor PI-103^15^—modulation of this signalling cascade does not exhibit an additive or synergistic effect when combined with temozolomide (TMZ), the standard chemotherapeutic agent for glioblastoma treatment. This holds true for both inhibition of PI3K directly or its downstream effector mTOR, with regards to clinical relevant parameters, apoptosis induction (Fig. 1d) and total cell number reduction (Fig. 1e).

Interestingly, clear differences between PI3K and mTOR inhibition became apparent when we investigated the potential role of this signalling cascade in motility. The results of the wound healing assay indicate that only GDC-0941 affects the cellular movement in this assay (Fig. 1f).

### The effects of PI3K and mTOR inhibition on primary glioblastoma populations

As established cell lines have been reported not to reflect the expression profiles of glioblastoma cell populations particularly well^24,25^, we switched to the previously described patient-derived G40 cell population^26^, which was also found to be negative for PTEN expression^18^. These cells, when propagated under stem cell-conditions (reduced serum conditions) express a stable phenotype similar to the expression profile observed in vivo^27^. Slow proliferating, Nestin-positive stem cell-like cells (SC) were induced to differentiate by the addition of serum to fast proliferating, GFAP-positive differentiated cells (DC)^15,26^. Importantly, while genetically identical to their parental SC population, the expression profile of the DC population is only considered stable short term, i.e. fresh DC populations were differentiated throughout the experiments^27^.

**Fig. 2:**
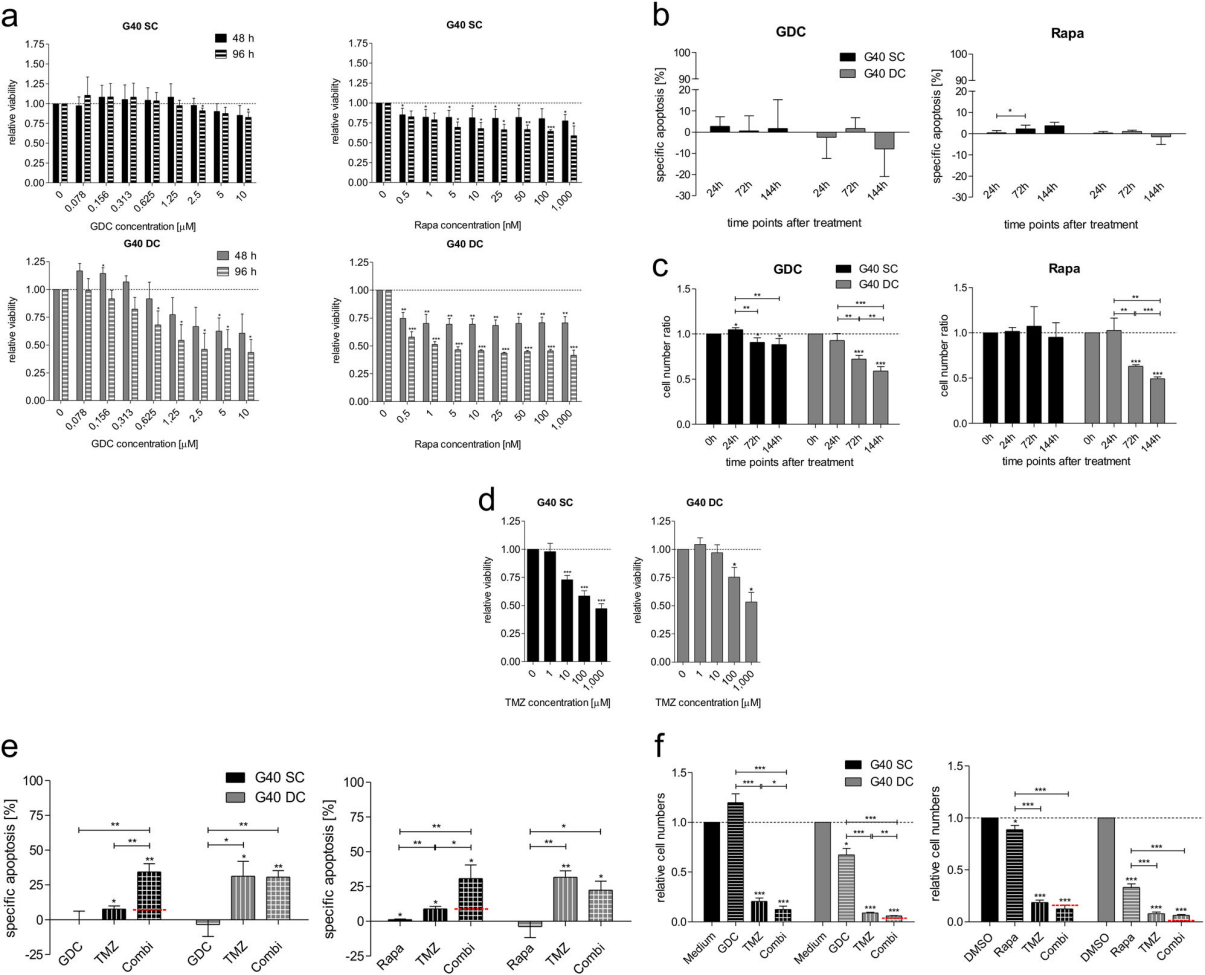
GDC-0941 (Pictilisib) and Rapamycin affect PI3K-mediated signalling in glioblastoma cell populations **a** The effect of the indicated concentrations of the two pharmacological inhibitors, GDC-0941 (GDC) left and Rapamycin (Rapa) right, on the viability of glioblastoma stem cells (SC; upper row) or differentiated cells (DC; lower row). Primary glioblastoma cell populations were isolated and maintained as previously described with approval of the Ethics Committee, Medical Faculty, Ulm University^26^. **b** Specific DNA fragmentation used as surrogate readout for apoptosis was assessed by flow cytometric analysis of propidium iodide-stained nuclei, for up to 144 h treatment with either 0.6 μM GDC-0941 (left), 5 nM Rapamycin (right). **c** Relative changes in cell numbers (control set at 1 for each individual time point), for up to 144 h treatment with either GDC-0941 (left) or Rapamycin (right). **d** G40 SC (left) and G40 DC (right) populations were treated with indicated concentrations of TMZ. After 144 h of treatment initiation cell viability was assessed using the MTT assay and data were normalised to the control treatment with DMSO only. **e** G40 SC and DC populations were incubated with either 0.6 μM GDC-0941 (left), 5 nM Rapamycin (right), 100 μM TMZ or the combination of inhibitor and TMZ. 144 h after treatment initiation DNA fragmentation, assessed by flow cytometric analysis of PI-stained cell nuclei, was used as readout for apoptosis. **f** G40 SC and DC populations were incubated with either 0.6 μM GDC-0941 (left) or 5 nM Rapamycin (right), 100 μM TMZ or the combination of inhibitor and TMZ. Total cell number was assessed 144 h after treatment initiation using the CASY cell counter. Cell numbers were normalised to the control. Experiments shown in **a** and **d** were performed at least three times in sextet, shown are mean and +SD, while **b**, **c**, **e** and **f** show results of at least three independent experiments performed in triplicate, shown are mean and +SD. Red dashed lines indicate the statistical value that defines the mean of an additive effect. Statistical significance of treatment substances was determined by one-sample *t* test, comparing the hypothetical value 1 (in **d** and **f**), or 0 (in **e**) and is indicated by asterisk above error bars, whereas statistical significance between two treatment substances was determined by unpaired two-tailed *t* test and is indicated by asterisks above the lines (*p < 0.05, **p < 0.01, ***p < 0.001)

G40 SC and DC populations were treated with GDC-0941 or Rapamycin and similar parameters were investigated as with the established cell lines (Fig. 2a–c). As with the GBM cell lines neither pharmacological inhibitors significantly induce cell death, but, interestingly, compared to the cell lines, primary material seems to be more responsive to Rapamycin than GDC-0941 and, with regards to metabolic activity and changes in cell numbers, stem cell-like cells appear more resistant to the effects of inhibiting the PI3K pathway than the DCs. This might be due to the fact that G40 SCs exhibit a lower rate of proliferation than G40 DCs^26^.

Next, we investigated the potential of PI3K and mTOR inhibition as a sensitiser for TMZ-induced apoptosis in primary material. When comparing the effects of various concentrations of TMZ on the metabolic activity of SC and DC populations, we found that—surprisingly—TMZ affected the metabolism of stem cell-like cells more strongly than that of their differentiated progeny (Fig. 2d). This is, however, not reflected in the apoptosis rates; here, it is clearly discernible that SCs are more chemoresistant than DCs (Fig. 2e). Interestingly, SCs can be sensitised for TMZ-induced cell death equally well by both substances, GDC-0941 and Rapamycin, increasing their apoptosis rate to that of DCs (Fig. 2e). The synergism in apoptosis induction is not clearly reflected in the reduction of total cell numbers, partially due to the strong effect of TMZ alone (Fig. 2f). These data suggest that the primary effect of TMZ on patient-derived glioblastoma cells might not be via apoptosis induction, but predominantly by inhibiting proliferation. TMZ has been described also as an inducer of autophagy by generating ROS and activating Erk, which antagonises the apoptotic effect^28–30^. Adding further pharmacological substances that act also predominately in an antiproliferative and autophagy-inducing fashion, such as inhibitors of PI3K signalling, will not further increase therapeutic efficacy^31^.

This is in line with our previous data showing that, in primary material, only rarely an additive or weakly synergistic effect can be seen between the dual-kinase inhibition and chemotherapy^15^. Furthermore, in terms of clinical applications, modulation of PI3K signalling has also not been successfully incorporated into targeted therapy for glioblastoma^7^, further highlighting the current failure of successful targeted approaches^1^.

However, that combining PI3K inhibition and TMZ sensitises SCs, but not DCs for apoptosis, raises the interesting possibility that the PI3K/Akt/mTOR pathway can have distinct functions in genetically identical, but diversely differentiated glioblastoma cells.

### Distinct functions for PI3K signalling in G40 stem cells and their differentiated progeny

The cancer stem cell hypothesis, which is not without controversy, postulates that even though cancer stem cells are not necessarily the drivers of tumourigenesis, a small, strongly therapy-resistant subpopulation of cells exists that, when transplanted in immunocompromised animal models at low numbers, initiate and maintain tumour xenografts that are similar to the parental tumour found in the patient^32,33^. Our own data strongly suggest that the situation is more complex in glioblastoma and that the stem cell phenotype is a consequence of providing a permissive environment^26^. However, we can show here that stem cell-like cells are more resistant to TMZ-induced apoptosis than their differentiated progeny (Fig. 2e), as is predicted by the tumour stem cell hypothesis^33^. Nevertheless there seems little doubt that different cell populations exist within a single tumour and we can show that genetically identical tumour cells can exhibit a different expression levels of components of the PI3K pathway^26^.

**Fig. 3:**
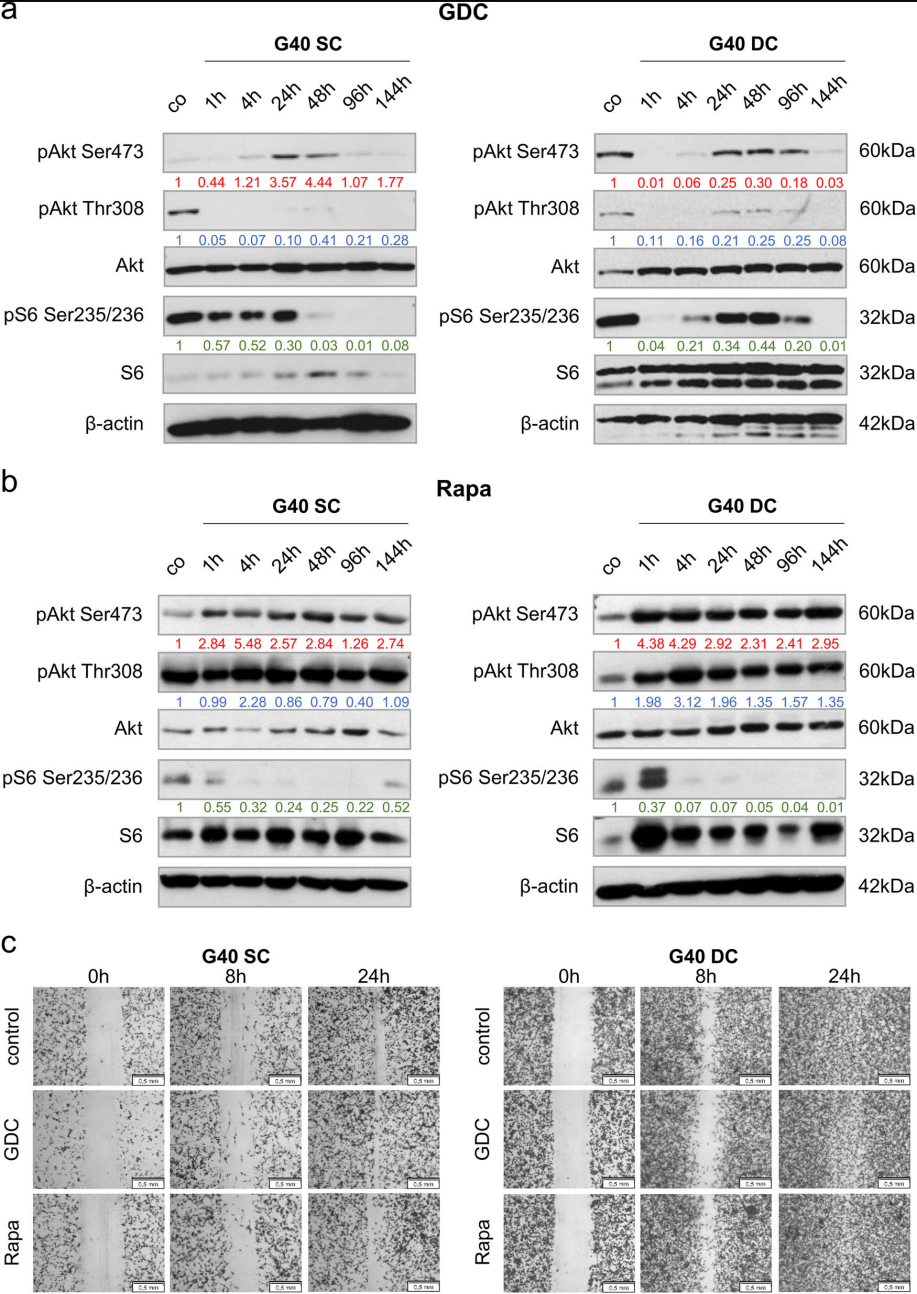
The regulation of PI3K signalling differs between stem cells and their differentiated progeny **a**, **b** The effect of the two pharmacological inhibitors on the PI3K signalling cascade, as assessed by Western blot analysis, using phosphorylation of Akt and S6, as surrogate readouts for PI3K and mTOR activity, respectively. Analysed were G40 glioblastoma stem cells (left) and differentiated cells (right) in the presence of either GDC-0941 **a** or Rapamycin **b**. Western blot analysis was performed as previously described^41^, and following antibodies were used: rabbit anti-phospho-AKT (Ser473) (#9271, Cell Signaling, Leiden, The Netherlands), rabbit anti-phospho-AKT (Thr308) (#9275, Cell Signaling), mouse anti-AKT antibody (#610860, BD Bioscience), rabbit anti-phospho-S6 ribosomal protein (Ser235/236) (#2211, Cell Signaling), rabbit anti-S6 ribosomal protein (#2317, Cell Signaling), β-actin (#A5441, Sigma-Aldrich) followed by goat anti-mouse IgG or goat anti-rabbit IgG-conjugated to horseradish peroxidase (Santa Cruz Biotechnology, Heidelberg, Germany). Of note, different exposure times were chosen between **a** and **b** to highlight the different effects of the two substances investigated. To facilitate a better comparison, relative protein phosphorylation was quantified using the ImageJ software packet (Rasband, W.S., ImageJ, U. S. National Institutes of Health, Bethesda, MD, USA, http://imagej.nih.gov/ij/, 1997–2011) and normalised to the control population. Red: phospho-Akt (Ser473)/Akt/ β-actin; blue: phospho-Akt (Thr308)/Akt/ β-actin; green: phospho-S6/S6/β-actin. **c** G40 SC (left) and DC (right) populations were incubated with either 0.6 μM GDC-0941 or 5 nM Rapamycin. Scratches were introduced 1 h after treatment initiation and from then semi-directional cell migration was examined after indicated time points by fixation of the cells and subsequent staining. Scale bars equal 500 μm. A representative result of at least two independent experiments is shown in **a** and **b**, while representative data of two independent experiments performed in triplicate are depicted in **c**

Therefore, we ascertained next whether both pharmacological inhibitors at the chosen concentrations specifically and lastingly inhibited their targets in SCs and DCs; for this we looked at phosphorylation of Akt and S6 as surrogate readouts for, respectively, PI3K and mTOR activity. At 0.6 μM GDC-0941 and 5 nM Rapamycin, both inhibitors are indeed specific: GDC-0941 affecting first Akt phosphorylation and only later/marginally S6 (Fig. 3a); while Rapamycin blocks phosphorylation of S6, which appears to cause a compensatory increase in phospho-Akt (Fig. 3b). Importantly, we could again observe differences between SC and DC populations: when treated with equal concentrations of GDC-0941 the downstream effectors of PI3K are differently affected: phospho-Akt to a lesser extent, although already in the untreated cells Akt seems less phosphorylated on Ser473 in the SCs than in the DCs (for a direct comparison^26^), and phospho-S6 rather strongly (Fig. 3a). Taken together with the previous data set, this clearly indicates that the PI3K/Akt/mTOR pathway is differently regulated and has different functions in genetically identical SC and DC populations. As we previously saw that blocking both PI3K and mTOR led to a reduction in DC movement^15^, a phenomenon we also observed with regards to the established cell lines (Fig. 1f), we next looked at cellular motility. Using a wound healing assay to induce semi-directional movement we observed no effect of either PI3K or mTOR inhibition on SC motility, while GDC-0941 appeared subtly to affect DC movement (Fig. 3c). To elucidate further the role of PI3K-mediated signalling in the SC and DC populations we focused on GDC-0941 which appeared to influence movement more strongly than Rapamycin.

### The role of PI3K signalling in motility and the therapeutic potential of GDC-0941

**Fig. 4:**
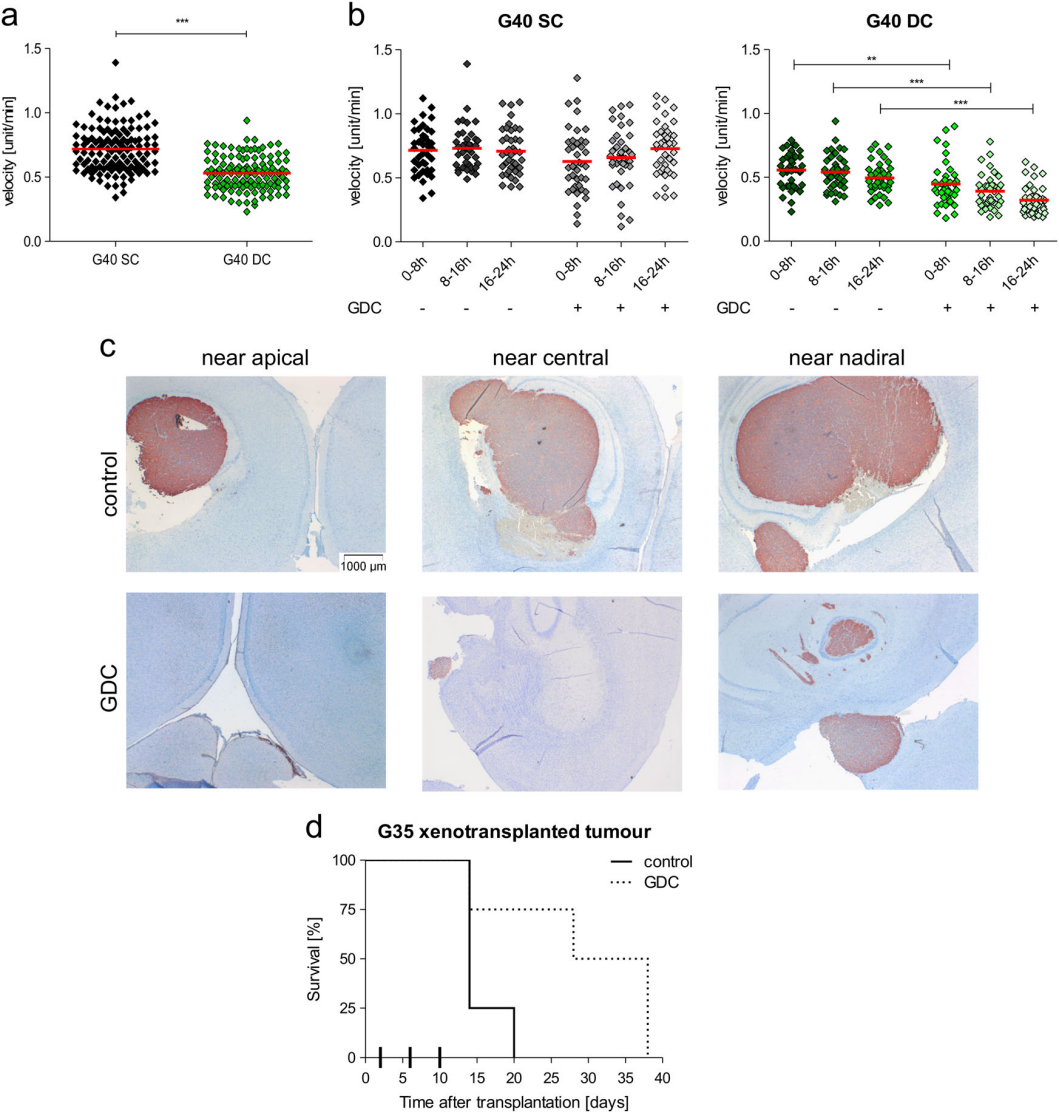
The velocity of different cell populations and how it is affected by GDC-0941 **a** The velocity of G40 SC and DC cell populations were analysed using ImageJ, as previously described^41^. Cells were allowed to grow for 24 h after seeding and were then recorded via time-lapse microscopy for another 24 h. Cell velocity was then examined in 8 h intervals. Mean of two experiments performed in duplicate is shown (*n* = 120). Statistical significance was assessed by unpaired two-tailed *t* test (***p < 0.001). **b** G40 SC (left) and DC (right) populations were allowed to grow for 24 h after seeding and were treated with GDC-0941 1 h before recording via time-lapse microscopy for another 24 h started. Cell velocity was examined in 8 h intervals using the software ImageJ. Mean of two experiments performed in duplicate is shown (*n* = 40). Statistical significance was assessed by two-way ANOVA (**p < 0.01, ***p < 0.001). **c**, **d** 1 × 10^5^ G35 stem cell-like cells per mouse were stereotactically implanted into the right striatum of immunocompromised mice, as previously described^41^. Mice were left untreated or treated with 15 mg/kg GDC-0941 at day 2, 6 and 10^18^. Animal experiments were approved by the Regierungspräsidium Tübingen, Germany. All animal experiments were conducted at animal houses at Ulm University. **c** Comparable sections through the brain of an untreated mouse and a mouse treated with GDC-0941. After preparing 3 μm slices from the whole mouse brain sections were processed as previously described^41^. Tissue was stained with hematoxylin and tumour cells were visualised by Vimentin staining (Abcam plc, Cambridge, UK), 14 days after implanting tumour cells and 4 days after the last of three treatments. Representative data from two treated and two untreated mice is shown (*n* = 4). The investigators were blinded while analysing the data and selecting representative samples per brain. **d** Survival time of *n* = 8 mice was evaluated and is shown here as Kaplan–Meier blot. The differences between untreated and treated are significant according to the Mantel–Cox test (*p* = 0.0446)

To verify the observation that PI3K signalling contributes to DC motility but not to the movement of SCs, we used a quantitative measurement and next looked at the cellular velocity. While stem cell-like cells exhibit a higher average speed than their progeny (Fig. 4a), inhibiting PI3K by GDC-0941 only affected the velocity of DCs. Here, it significantly retarded movement (Fig. 4b). Both observations are of high interest: while the difference in velocity between SC and DC populations is relatively minor—SCs are 10–20% faster than DCs—it is tempting to speculate that increased speed might contribute to increased invasion. Glioblastoma is a highly invasive tumour, and individual invasive cells or cell aggregations, even in the absence of a distinct tumour mass, are sufficient to cause neurological symptoms and death^34^. Furthermore, the almost systemic nature of the disease prevents complete surgical resection^35,36^. It has previously been suggested that the C-X-C chemokine receptor type 4 and its ligand contribute to glioblastoma invasion and are upregulated in stem cell-like cells^33^ and that the highly motile phenotype of glioblastoma cells is linked to reduced proliferation, the so-called 'the cost of migration' or 'go or grow' dilemma^37,38^. Whether the increased velocity of SCs is a consequence of their reduced proliferation compared to DCs^26^, or an additional independent feature mediating glioblastoma invasion remains to be elucidated. Supporting the latter possibility is the fact that PI3K signalling contributes to the motility of DCs but not SCs, suggesting a different underlying locomotive engine.

Finally, to verify that cell culture conditions did not unduly enhance the role of PI3K signalling in glioblastoma, we chose an in vivo setting that provided a structural microenvironment in the form of an orthotopic mouse model. Although G40 cells behave highly invasively when implanted into the brains of immunosuppressed mice, they form no discernible tumour bulk^33^. Therefore, G35 cells were selected, which are also negative for PTEN expression and show a similar profile when exposed to GDC-0941 as G40 (Supplementary Fig. 1) and, when implanted into a murine brain, form a tumour bulk with invasive edges and satellite cell aggregates throughout the brain^18,26^. Treating glioblastoma-bearing mice with GDC-0941 led to an apparent reduction of tumour volume but still resulted in a multifocal tumour (Fig. 4c). This is in accord with our in vitro data, assuming that a tumour consists predominantly of DCs and that invasion is mediated via stem cell-like cells. Both assumptions are accepted for many cancers, including glioblastoma^33^. Alternatively the presence of clusters of individual cells or small cell aggregates might be due to the potentially reduced ability of GDC-0941 to penetrate an intact blood-brain-barrier. It has been reported that high concentrations of the drug are only found within the tumour bulk, and that it is less well distributed throughout the healthy brain, i.e. where the invading cells are^39^. Treatment with GDC-0941 significantly prolongs survival of the mice (Fig. 4d), suggesting a therapeutic potential of targeting PI3K signalling in glioblastoma. However, the target within this signalling network has to be chosen carefully, as our previous data show that replacing Rapamycin with GDC-0941 in a complex combination therapy can actually ablate the treatment's efficacy, as the latter inhibitor also reduces tumour vascularisation and thus blocks the effective delivery of chemotherapeutica^18^.

Taken together, as far as we are aware our data are the first clearly to demonstrate that within a glioblastoma tumour that harbours increased activation of PI3K signalling the PI3K pathway has distinct roles in different, but genetically identical cell populations. The pathway does not mediate increased resistance to therapy per se, but significantly contributes to the increased apoptosis resistance of SCs compared to DCs. The therapeutic use of pharmacological inhibitors of the PI3K pathway needs to be carefully evaluated as it is associated with an increase in cell numbers in the DC population and in glioblastoma cell lines, most likely by mediating proliferation. Blocking proliferation can reduce cellular sensitivity towards chemotherapy. This is in line with our previous data in both neuroblastoma and glioblastoma demonstrating that the temporal sequence of applying pharmacological inhibitors of PI3K signalling and chemotherapy has to be carefully considered^23^. Proliferation of the SC population is less affected by inhibition of PI3K signalling, which might in part be due to the fact that SCs proliferate more slowly than DCs. However, as G40 SCs proliferate much closer to the rate of G40 DCs than is the case in the G35 populations^26^, this is unlikely to be the whole explanation. While therapy-resistance and proliferation seem to be mediated via mTOR, the effect on DC motility is most likely mediated by a different side arm of the PI3K network, as Rapamycin affected semi-directed movement less strongly than GDC-0941. In conclusion when considering tumour-specific alterations in signalling networks, particularly in the context of potential therapeutic interventions, it is not sufficient to look at the genetic profile of the single cell population within the tumour. Signalling cascades can have distinct roles within different, but genetically identical cell populations that make up an individual tumour.

## Supplementary information

Suppl Fig S1
Suppl Fig S1
