# Supplementary material for: The effects of PI3K-mediated signalling on glioblastoma cell behaviour
Source: Oncogenesis. 2017 Nov 29;6(11):398. doi: 10.1038/s41389-017-0004-8 (PMC5868055; doi:10.1038/s41389-017-0004-8)
Supplement: Supplementary file 1 — Suppl Fig S1 [file 41389_2017_4_MOESM1_ESM.doc]

**Supplementary Figure S1: The effects of GDC-0941 (Pictilisib) on G35 cell populations**

(a): The effect of the indicated concentrations of GDC-0941 on the viability of glioblastoma stem cells, G35 SC (left), or differentiated cells, G 35 DC (right).

(b): Specific DNA fragmentation used as surrogate readout for apoptosis as assessed by flow cytometric analysis of propidium iodide-stained nuclei, for up to 144 hrs treatment with 0.6μM GDC-0941.

(c): Relative changes in cell numbers (control set at 1 for each individual time point), for up to 144 hrs treatment with GDC-0941.

(d): The effect of the pharmacological inhibitor GDC-0941 on the PI3K signalling cascade, as assessed by Western blot analysis, using phosphorylation of Akt and S6, as surrogate readouts for PI3K and mTOR activity, respectively. Analysed were G35 glioblastoma stem cells (left) and differentiated cells (right).

(e): G35 SC (left) and DC (right) populations were incubated with 0.6μM GDC-0941. Scratches were introduced 1hr after treatment initiation and from then semi-directional cell migration was examined after indicated time points by fixation of the cells and subsequent staining. Scale bars equal 500μm.

Experiments shown in (a) were performed at least three times in sextet, shown are mean and +SD, while (b) and (c) show results of at least three independent experiments performed in triplicate, shown are mean and +SD. In (d) and (e) exemplary results of at least two independent experiments are shown. Statistical significance of treatment substances was determined by one-sample t-test, comparing to the hypothetical value 1 [in (c)] and is indicated by asterisk above error bars whereas statistical significance between two treatment substances was determined by unpaired two-tailed t-test and is indicated by asterisks above the lines (*p<0.05, **p<0.01, ***p<0.001).
