# Supplementary figures and images for: The effects of PI3K-mediated signalling on glioblastoma cell behaviour
Source: Oncogenesis. 2017 Nov 29;6(11):398. doi: 10.1038/s41389-017-0004-8 (PMC5868055; doi:10.1038/s41389-017-0004-8)

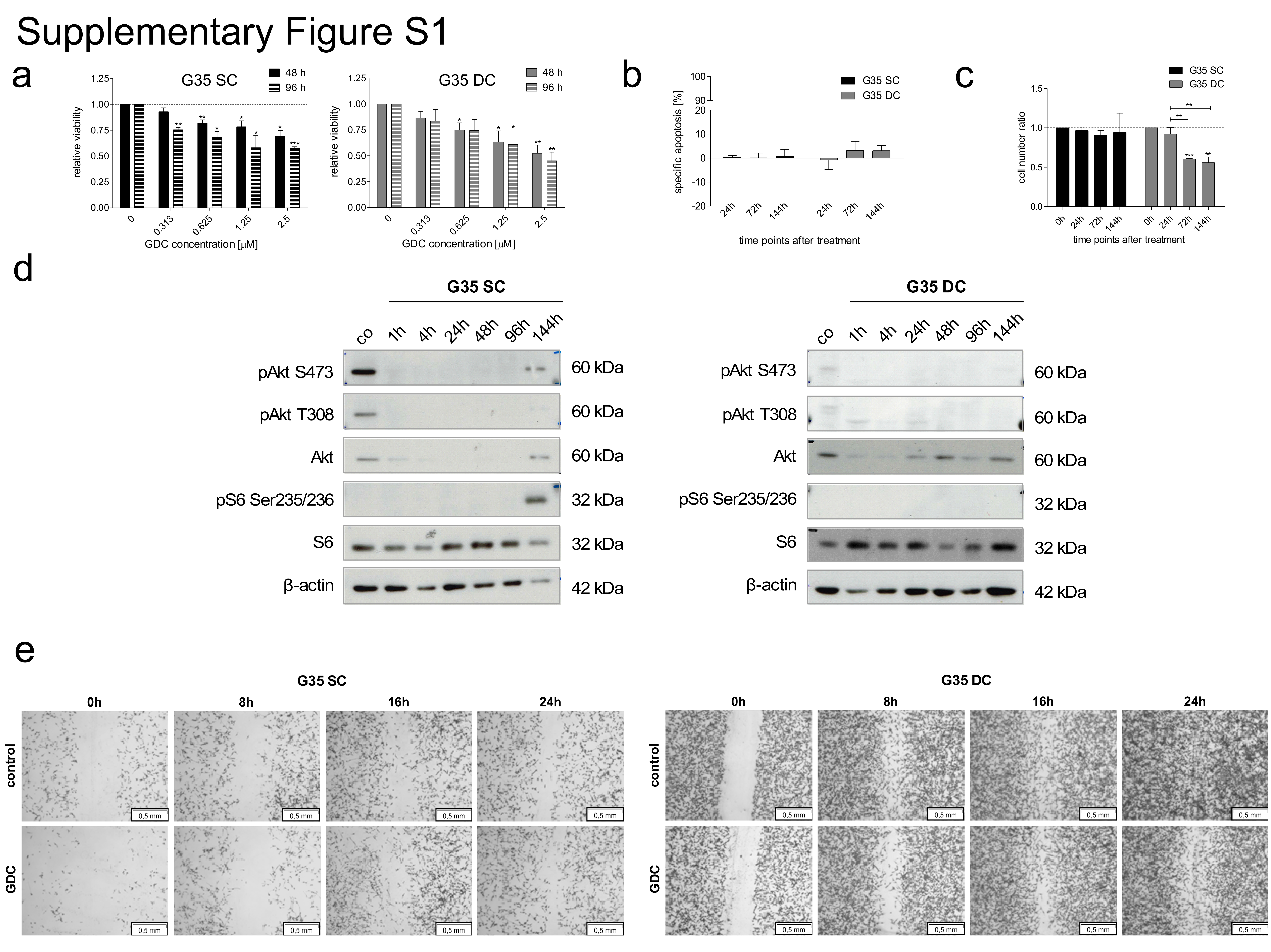

Supplement: Supplementary file 2 — Suppl Fig S1 [file 41389_2017_4_MOESM2_ESM.tif]
